# ASO Author Reflections: Closing the Staging Gap in Gallbladder Cancer: Preoperative Risk Enrichment for Occult Nodal Disease

**DOI:** 10.1245/s10434-026-19509-0

**Published:** 2026-03-21

**Authors:** Jun Kawashima, Itaru Endo, Timothy M. Pawlik

**Affiliations:** 1 Department of Gastroenterological Surgery, Yokohama City University School of Medicine, Yokohama, Japan; 2 Department of Surgery, The Ohio State University Wexner Medical Center and James Comprehensive Cancer Center, Columbus, OH USA

## Past

In gallbladder cancer (GBC), lymph node metastasis is associated with poor outcomes, even after curative-intent resection.^1^ Despite advances in cross-sectional and functional imaging—including ultrasound, computed tomography, magnetic resonance imaging, and positron emission tomography—accurate preoperative identification of nodal disease remains challenging.^2^ In this setting, occult nodal disease (OND), defined as pathologically confirmed nodal metastasis in patients without clinical or radiographic evidence of lymph node involvement, represents a clinically important concept.^3^ However, the prevalence and determinants of OND in GBC have been poorly characterized, despite the well-established prognostic significance of nodal metastasis. As a result, some patients undergo resection under the assumption of node-negative disease only to be upstaged on final pathology, underscoring the need for better preoperative risk stratification.

## Current

To address this clinical gap, we leveraged an international, multi-institutional cohort spanning both Western and Eastern centers to characterize OND in GBC and to identify readily available preoperative markers that refine risk stratification and inform clinical decision-making.^4^ Among 142 patients classified as clinically node-negative (cN0), 47 (33.1%) had pathologic nodal metastasis on final evaluation—consistent with OND. On multivariable analysis, preoperative jaundice, higher carbohydrate antigen 19-9 (CA19-9), and a higher Systemic Immune-Inflammation Index (SII) were each independently associated with OND. Notably, the incidence of OND increased stepwise with the number of elevated markers: 17.1% among patients with no markers, 44.8% among those with one marker, and 73.6% among patients with two to three markers. Collectively, these findings highlight the potential of simple, routinely available clinical and laboratory measures to enrich risk for OND and to support pragmatic preoperative risk stratification in GBC.

## Future

Incorporating preoperative jaundice, CA19-9, and SII into routine assessment may help identify clinically node-negative patients at high risk of OND. These patients may benefit from intensified regional nodal evaluation, either preoperatively with endoscopic ultrasound and/or with lymphadenectomy at the time of hepatic resection. Patients at the highest risk of OND may represent a subgroup with biologically aggressive disease who may benefit from alternative treatment sequencing, including systemic therapy before surgery.^5^ Future work should focus on external and prospective validation of these markers and on evaluating integrated staging pathways that combine imaging, serologic markers, and minimally invasive diagnostics.
